# Clinical Progress in Mesenchymal Stem Cell Therapy: A Focus on Rheumatic Diseases

**DOI:** 10.1002/iid3.70189

**Published:** 2025-05-12

**Authors:** Helal F. Hetta, Alaa Elsaghir, Victor Coll Sijercic, Abdulrahman K. Ahmed, Sayed A. Gad, Mahlet S. Zeleke, Fawaz E. Alanazi, Yasmin N. Ramadan

**Affiliations:** ^1^ Division of Microbiology, Immunology and Biotechnology, Department of Natural Products and Alternative Medicine, Faculty of Pharmacy University of Tabuk Tabuk Saudi Arabia; ^2^ Department of Microbiology and Immunology, Faculty of Pharmacy Assiut University Assiut Egypt; ^3^ North Central College Naperville Illinois USA; ^4^ Emergency Medicine Unit, Department of Anaethesia and Intensive Care, Faculty of Medicine Assiut University Assiut Egypt; ^5^ Menelik II Medical and Health Science College Addis Ababa Ethiopia; ^6^ Department of Pharmacology and Toxicology, Faculty of Pharmacy University of Tabuk Tabuk Saudi Arabia

**Keywords:** mesenchymal stem cells, osteoarthritis, rheumatic diseases, rheumatoid arthritis, systemic lupus erythematosus, systemic sclerosis

## Abstract

**Background:**

Rheumatic diseases are chronic immune‐mediated disorders affecting multiple organ systems and significantly impairing patients' quality of life. Current treatments primarily provide symptomatic relief without offering a cure. Mesenchymal stem cells (MSCs) have emerged as a promising therapeutic option due to their ability to differentiate into various cell types and their immunomodulatory, anti‐inflammatory, and regenerative properties. This review aims to summarize the clinical progress of MSC therapy in rheumatic diseases, highlight key findings from preclinical and clinical studies, and discuss challenges and future directions.

**Methodology:**

A comprehensive review of preclinical and clinical studies on MSC therapy in rheumatic diseases, including systemic lupus erythematosus, rheumatoid arthritis, ankylosing spondylitis, osteoarthritis, osteoporosis, Sjögren's syndrome, Crohn's disease, fibromyalgia, systemic sclerosis, dermatomyositis, and polymyositis, was conducted. Emerging strategies to enhance MSC efficacy and overcome current limitations were also analyzed.

**Results and Discussion:**

Evidence from preclinical and clinical studies suggests that MSC therapy can reduce inflammation, modulate immune responses, and promote tissue repair in various rheumatic diseases. Clinical trials have demonstrated potential benefits, including symptom relief and disease progression delay. However, challenges such as variability in treatment response, optimal cell source and dosing, long‐term safety concerns, and regulatory hurdles remain significant barriers to clinical translation. Standardized protocols and further research are required to optimize MSC application.

**Conclusion:**

MSC therapy holds promise for managing rheumatic diseases, offering potential disease‐modifying effects beyond conventional treatments. However, large‐scale, well‐controlled clinical trials are essential to establish efficacy, safety, and long‐term therapeutic potential. Addressing current limitations through optimized treatment protocols and regulatory frameworks will be key to its successful integration into clinical practice.

## Graphical abstract

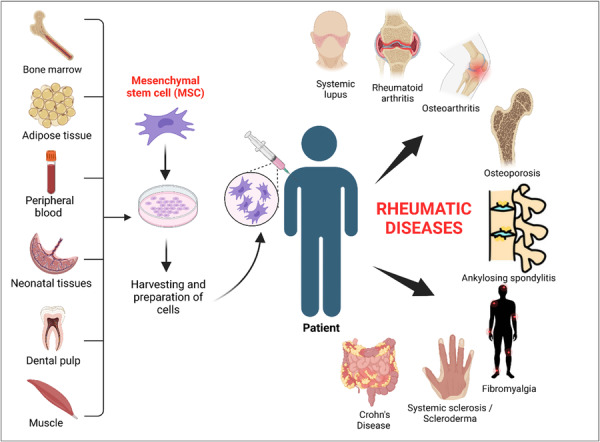

## 1 Introduction

Stem cells are a group of undifferentiated cells that are distinguished by their capacity to divide indefinitely (self‐renewal), often originate from one particular cell (clonal), and can develop into a wide variety of distinct cell kinds and sorts of tissues (potent). There are many different sources of stem cells, each of which has a unique potency. Pluripotent cells are embryonic stem cells (ESCs), which come from the blastocyte, the innermost layer of cells in an embryo. In contrast, induced pluripotent stem cells (iPSCs) come from somatic cells that have been reprogrammed. Pluripotent stem cells (PSCs) have the ability to develop into tissue derived from any of the three layers of germ cells, which are endoderm, mesoderm, and ectoderm. Multipotent stem cells develop into tissues that originate from only one germ layer. One example of this is the formation of mesenchymal stem cells (MSCs), which are responsible for the formation of bone, adipose, and cartilage structures [1].

**Figure 1:**
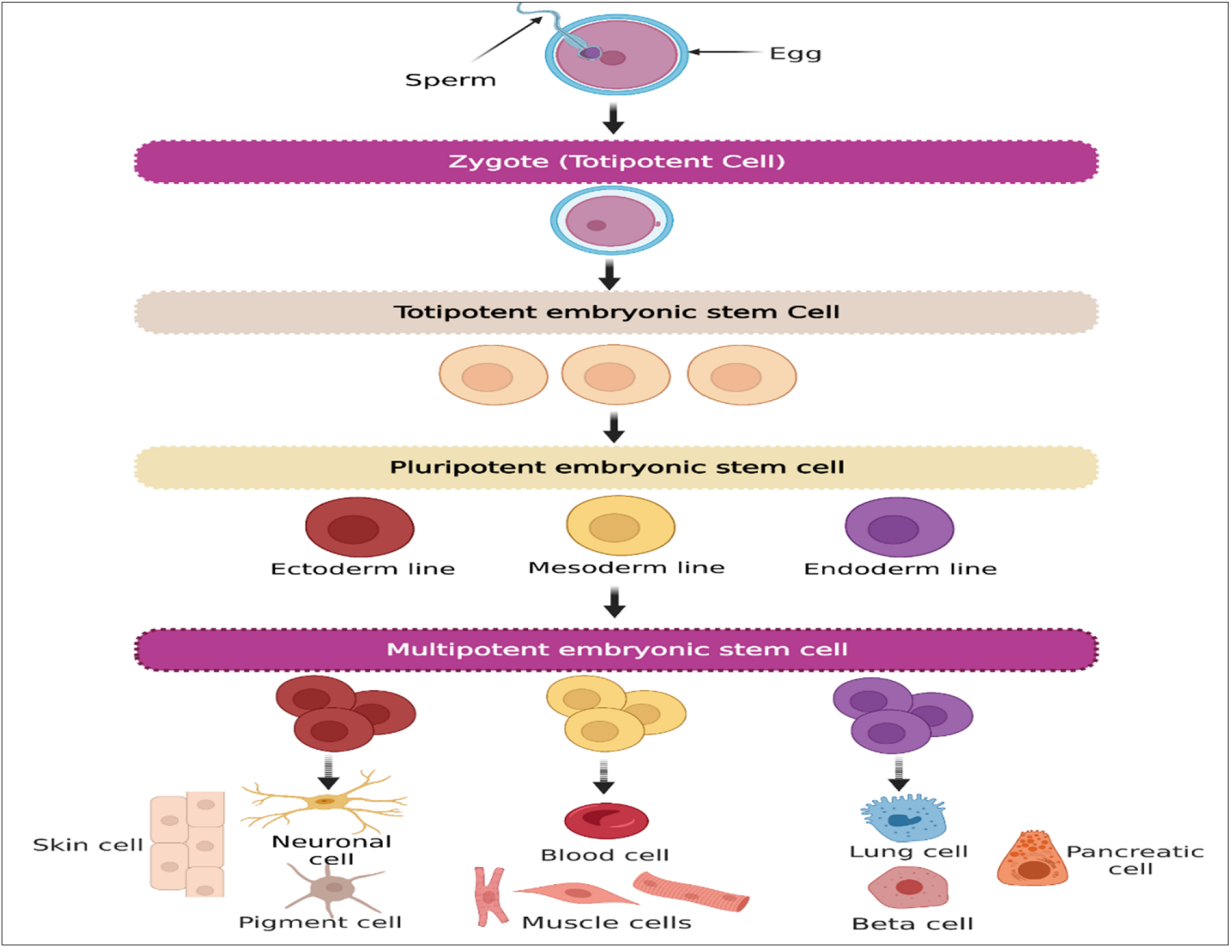
Different types of stem cells. Totipotent, Pluripotent, and Multipotent stem cells. Totipotent stem cells are produced after fertilization and zygote development. They can differentiate into almost any kind of body cell or tissue, including the placenta. Pluripotent stem cells are embryonic stem cells with the ability to proliferate, self‐renew, and develop into cells of the early primary germ cell layers, namely mesoderm, endoderm, and ectoderm. Multipotent stem cells develop into tissues that originate from only one germ layer. For example, neuron cells and skin cells originate from ectoderm, blood cells and muscle cells originate from mesoderm, and lung cells, and pancreatic cells originate from endoderm.

Based on their ability to differentiate into other cell types, stem cells are classified as either pluripotent, multipotent, omnipotent, totipotent, oligopotent, or unipotent [2]. Depending on where they originated from, stem cells may be classified as embryonic, adult, fetal, or iPSCs [3, 4]. Generally, ESCs and iPSCs have pluripotent properties, but adult stem cells (ASCs) either have unipotent or oligopotent properties [1]. Multipotent cells that can reproduce and replace damaged or dead cells in the body are called MSCs. Additionally, MSCs produce immunomodulatory molecules, resulting in the development of an environment that is highly conducive to tissue regeneration. They are found in a variety of tissues and organs, including bone marrow (BM), adipose tissue, skin, fallopian tube, umbilical cord (UC), liver, and lungs (Figure 1). According to growing evidence, MSCs provide a promising option for cell therapy and rebuilding of human tissues owing to their self‐renewal capacity, differentiation multipotency, paracrine potentials, long‐term ex vivo proliferation, as well as immunomodulatory effects [5]. Additionally, MSCs can aid in the development and differentiation of additional stem cells. They have the ability to release bioactive molecules, which are crucial for tissue regeneration [6, 7, 8]. These characteristics lead to advancements in the treatment of a variety of illnesses, including those that affect the bones, neurons, lungs, liver, heart, kidney, etc [6].

Rheumatic diseases are an umbrella word that refers to arthritis in addition to other conditions that affect joints, tendons, muscles, ligaments, bones, and muscles [9]. Rheumatic diseases are a collection of immune‐mediated and inflammation‐related disorders with a wide variety of clinical presentations that may affect every system in the body, not just the musculoskeletal one. It is thought that genetic susceptibility, environmental factors, immune system problems, abnormal cell death, reactive oxygen species, and excess autoantibodies all play a part in the development of these disorders [10, 11]. Rheumatoid arthritis (RA), systemic lupus erythematosus (SLE), and systemic sclerosis (SSc) are examples of rheumatic illnesses, which are inflammatory syndromes caused by immune system dysregulation [12]. These conditions can affect multiple organs and tissues in the body, leading to chronic pain, joint stiffness, fatigue, irreversible disability, and organ damage. They are often characterized by the presence of autoantibodies and can have a significant impact on a person's quality of life [10, 12, 13].

The need to reestablish immunologic self‐tolerance to achieve long‐lasting remissions or stimulate tissue regeneration has been the driving force behind the development of cellular treatments. In recent years, therapies centered around MSCs have shown promise in the management of rheumatic diseases [13]. The contribution of stem cells to modern medicine is of paramount importance, both for their broad use in basic research and for the opportunities they give us to develop new therapeutic strategies in clinical practice [14]. Their characteristics make them valuable in a wide range of applications in biological and medical sciences [15]. In this article, we highlight recent developments and state‐of‐the‐art information about the clinical and therapeutic applications of MSCs in a variety of rheumatic illnesses.

## 2 Stem Cells in Regenerative Medicine

Numerous investigations over the past few years have shown that cellular treatment has advanced significantly in both in vitro and in vivo studies. Stem cells are vital for physiological regeneration since they can regenerate themselves and differentiate into any type of cell. Regarding tissue regeneration, there are several sources of PSCs, ASCs, iPSCs, and ESCs [16]. Because of their strong capacity for self‐renewal and pluripotency, PSCs are a significant option for managing several disease issues. Yet, utilizing these cells raises ethical concerns since embryos must be destroyed because ESCs are extracted from blastocyst‐stage embryos [17, 18, 19]. Preclinical research has demonstrated the capacity of iPSCs to regenerate tissue, and the first clinical trial to treat age‐related macular degeneration has been conducted [20, 21]. However, the risk of tumor development is still a mystery. Due to these drawbacks, scientists started looking into ASCs, which are multipotent stem cells present in adult organs and tissues. According to several studies, stem cell treatment may regenerate and repair damaged organs and tissues, including bone regeneration, osteoarthritis (OA), pulpitis, ischemic heart tissue, wound healing, cutaneous healing, Type 1 diabetes, and foot ulcers [6, 22, 23, 24, 25, 26, 27, 28, 29, 30, 31, 32]. Furthermore, several studies have shown that cultivated ASCs produce a variety of molecules that promote regeneration and possess immunoregulatory, anti‐apoptotic, chemoattractant as well as angiogenic properties [27, 33, 34]. The most popular ASCs are hematopoietic stem cells (HSCs) and MSCs, which can be obtained even from diseased individuals.

### 2.1 Mesenchymal Stem Cells

MSCs were initially identified as multipotent stem cells by Friedenstein and colleagues at the end of 1960 [35]. Non‐hematopoietic MSCs can develop into a variety of lineages, such as chondrocytes, osteocytes, and adipocytes in the mesoderm, neuroblasts in the ectoderm, and hepatocytes in the endoderm [36, 37]. MSCs were formerly believed to be “stromal” cells rather than stem cells [38]. Several researchers attempted to change the nomenclature of MSCs to medicinal signaling cells owing to their role in the release of certain metabolic molecules at the inflamed, injured, and diseased sites [39, 40]. Later on, some research investigated that MSCs may produce prostaglandin E2 (PGE2), which is crucial for MSCs' ability to self‐renew, modulate the immune system, and trigger a series of actions that prove their stemness. As a result, the name “mesenchymal stem cells” is warranted [41].

MSCs are mostly located in the BM, and they exhibit multilineage differentiation as well as the capacity for self‐renewal [18, 42, 43]. They could be extracted from a variety of organs and tissues, such as BM, adipose tissue, peripheral blood, Wharton's jelly, dental pulp, amniotic fluid, placenta, and UC [44, 45, 46]. However, according to a recent review, the most prevalent and commonly utilized adult donor tissues for human MSCs are BM and the stromal vascular part of adipose tissue [47]. Depending on the source of separation, MSCs can exhibit a variety of surface markers and cytokine profiles. The most prevalent markers that characterize MSC are CD73, CD105, and CD90, with no expression of CD11b, CD14, CD19, CD34, CD45, CD79, or HLA‐DR [48, 49, 50, 51]. The MSCs possess a variety of characteristics that make them an ideal source for cell therapy, including stemness potency, ease of isolation from various sources, rapid expansion in large quantities for clinical use, fewer ethical concerns than ESCs, a lower risk of teratoma formation than iPSCs, and benefits for a wide range of therapeutic applications because of their ability to migrate to injured tissue through chemo‐attraction [52, 53, 54]. Additionally, MSCs have the capacity to produce a range of bioactive substances such as growth factors, proteins, chemokines, cytokines, and microRNAs which may indicate appropriate applications for them [55].

### 2.2 Biological Roles of MSCs

In inflammatory cytokine‐rich conditions, such as wounds, infections, or immunological‐mediated diseases, MSCs have the capacity to suppress the immune response. These MSC immunoregulatory characteristics include inducing the M1 to M2 macrophage transition and inhibiting T cell activation and proliferation [56, 57, 58]. This distinct behavior of MSCs in either the presence or absence of inflammatory mediators is known as MSC polarization. Furthermore, after systemic treatment, MSCs can migrate to damaged regions and later exhibit therapeutic activity through a number of mechanisms, most notably angiogenesis and immunoregulation [56, 59]. While the immunosuppression mechanism of MSC is not fully understood, it seems that cellular contact, combined with several factors plays the primary role in this mechanism. Rheumatic diseases characterized by high levels of inflammatory cytokines, such as interferon‐gamma (IFN‐γ) and tumor necrosis factor‐alpha (TNF‐α) which stimulate MSCs to produce various kinds of cytokines, such as hepatocyte growth factor (HGF) and transforming growth factor‐beta (TGF‐β), soluble factors like PGE2, indoleamine‐2,3‐dioxygenase (IDO), and nitric oxide (NO). To maximize their immunomodulatory effects, these mediators inhibit effector T cells and boost the expression of FOXP3, GITR, and CTLA4 in regulatory T cells (Tregs) [60, 61, 62].

**Figure 2:**
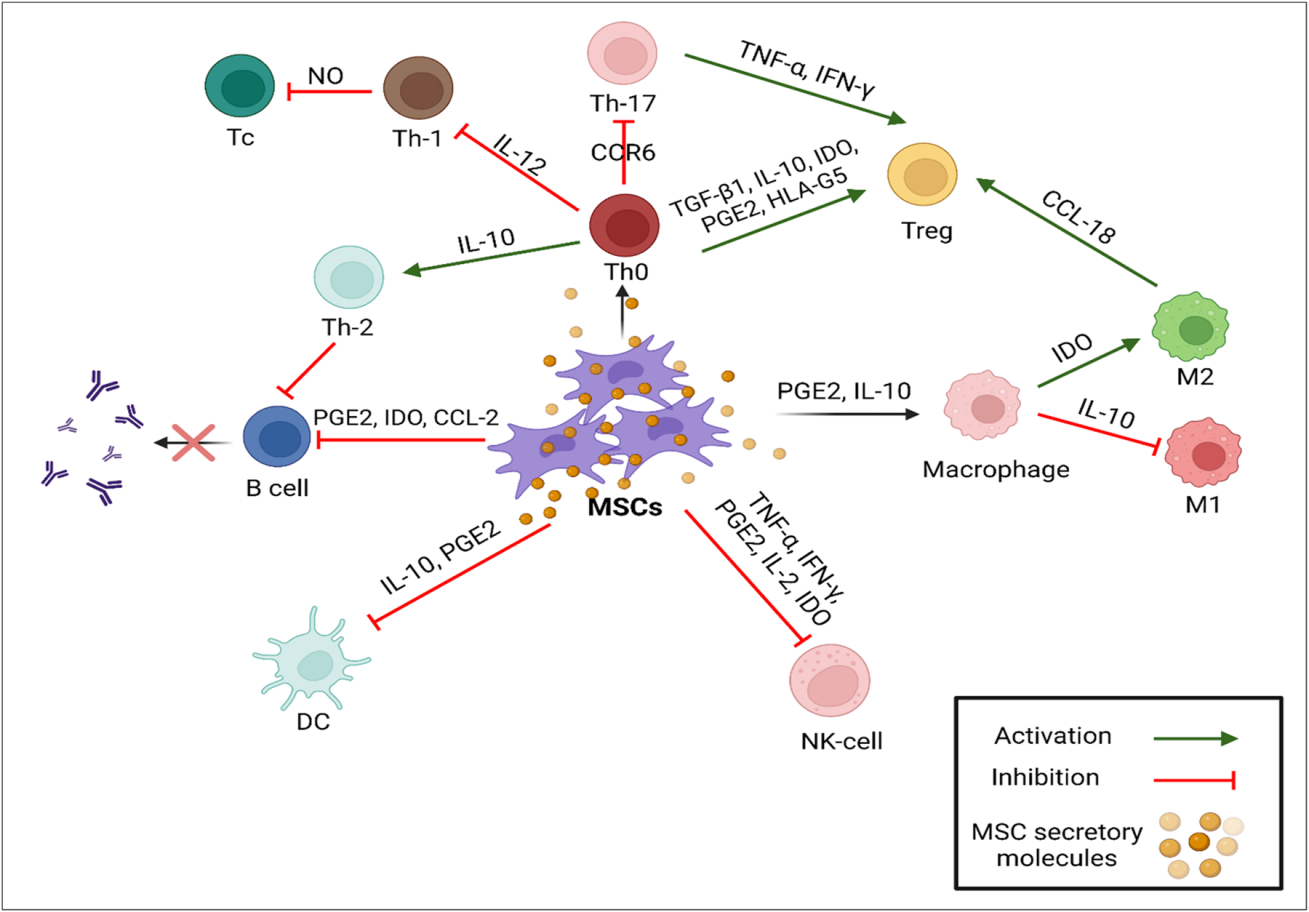
Immunomodulatory effects of MSCs. MSCs exert immunomodulatory properties via direct contact with immune cells, as well as secretory molecules generated by MSCs' paracrine mechanism. MSCs mainly have immunosuppressive effects, although positively regulate and activate Th2 differentiation, Treg cell production, and M2 macrophage differentiation.

Additionally, cell‐to‐cell contact makes it easier for cytokine‐primed MSCs to stimulate Tregs. Effective Tregs are stimulated by the overexpression of inducible co‐stimulator ligands (ICOSL) [63]. MSCs can also indirectly improve the production of Treg cells. According to previous in vitro studies, MSCs activate M2 macrophage and change the phenotype via secreting extracellular vesicles [64]. Additionally, after being stimulated by MSCs, M2 macrophages express C‐C motif chemokine ligand (CCL‐18) and produce Treg cells [65]. Additionally, MSCs boost the production of cyclooxygenase 2 (COX2) and IDO, which causes M2 cells to produce CD 206 and CD163, as well as interleukin (IL)‐6 and IL‐10 in the microenvironment [66]. When MSCs are co‐cultured, the upregulation of IL‐10 generated by dendritic cells (DCs) and M2 cells results in additional immunomodulation through inhibiting effector T cells [67, 68]. Moreover, IDO secreted by MSCs can inhibit B cell activation, expansion, and IgG release, which inhibits T effector cells [69, 70] (Figure 2).

**Figure 3:**
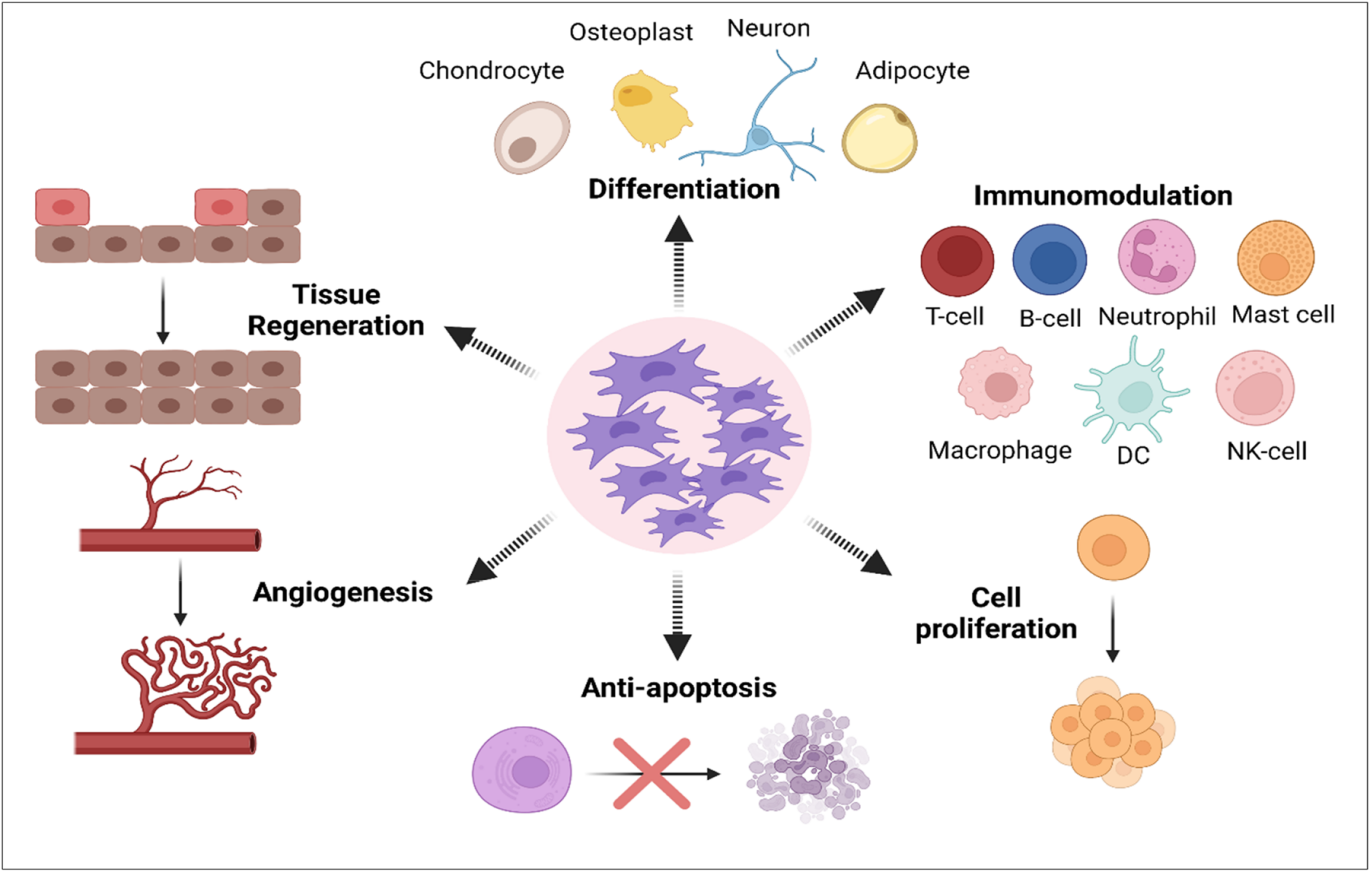
Biological roles of mesenchymal stem cells (MSCs). MSCs have been employed in recent research as therapeutic candidates as they possess immunomodulatory effects, can differentiate into different cell types, have antifibrotic activity enhance tissue regeneration and cell proliferation, promote angiogenesis, and help in the restoration of tissue function.

Considering that MSCs can differentiate into various types of cells, exhibit antifibrotic activity, boost angiogenesis and tissue regeneration, and support the restoration of tissue function, they have been used as therapeutic options (Figure 3). One of MSCs' defining characteristics is their multipotency, which allows them to differentiate in vitro into several types of tissues. In vitro, chondrogenic differentiation of MSCs is frequently achieved by cultivating them in the presence of TGF‐β1 or TGF‐β3, insulin‐like growth factor‐1 (IGF‐1), fibroblast growth factor 2 (FGF‐2), or bone morphogenetic protein 2 (BMP‐2) [71, 72, 73]. MSC development into chondroblasts is marked by an increase in the expression of numerous genes such as collagen Type II, IX, aggrecan, and chondroblast cell shape. During the chondrogenesis process, FGF‐2 stimulates MSCs produced by TGF‐β1, TGF‐β3, and/or IGF‐1 [74]. Numerous molecular pathways, including TGF‐βs, hedgehog, Wnt/‐catenin, FGFs, and BMPs have been shown in studies to control chondrogenesis [75]. Additionally, MSCs have the ability to perform Osteogenesis activity by being stimulated by vitamin D3, β ‐glycerophosphate, ascorbic acid, and/or BMP‐2, BMP‐4, BMP‐6, and BMP‐7 [76].

MSCs have anti‐fibrotic action as one of their main characteristics. These cells can develop both in vivo and in vitro into a variety of cell lineages, like hepatocytes [77]. Numerous trophic factors included in MSCs encourage progenitor cells and matrix remodeling to aid in the repair of damaged cells. MSCs can reduce myofibroblasts and stop fibrotic activity in damaged tissues [78]. Additionally, these cells produce pro‐angiogenic substances including vascular endothelial growth factor (VEGF), IGF‐1, and anti‐inflammatory substances that aid in the restoration of tissue function. For example, in an animal model of heart illness, MSCs can promote neovascularization of ischemic myocardium via VEGF [79]. Additionally, IGF‐1 has a positive impact on the proliferation and survival of cardiomyocytes [80].

## 3 Stem Cell Therapy in the Treatment of Different Rheumatic Diseases

### 3.1 MSCs in SLE

SLE is a persistent immune‐mediated illness that is defined by the emergence of various self‐antibodies, particularly antinuclear antibodies, and the involvement of many body systems. In the etiology of SLE, it has been demonstrated that both genetic predisposition and environmental variables, including medicines, UV radiation, infection, and anxiety, play important roles [81]. The condition affects people of all racial and ethnic backgrounds, although it is more prevalent among black populations and females [82]. Antigen‐antibody complexes are formed during an episode of SLE and then migrate across multiple organs and tissues, including the basement membrane of the skin, the kidneys, and numerous other parts of the body [83]. Stimulation of B‐lymphocytes, generation of self‐antibodies, and development of immune system complexes are all part of the process of SLE, whatever the reasons that cause it [84].

The manifestations of SLE might vary from person to person but might involve things like general exhaustion, rashes on the skin, a high body temperature, and joint discomfort or swelling. In some individuals, the onset of SLE manifestations, known as flares, may occur at irregular intervals, sometimes years apart, and then disappear during periods known as remissions. It's possible, nevertheless, that some people with SLE will have more severe flares. Sun sensitivity, mouth ulcers, arthritis, lung issues, cardiovascular issues, renal issues, convulsions, mental disorders, and abnormalities in blood cells and the immune system are other potential manifestations [85, 86, 87].

There is currently no one‐size‐fits‐all therapy or cure for SLE. Many patients have unwanted side effects from modern therapies such as antimalarial, immunosuppressant, and glucocorticoid medications. Even with the availability of treatment options, many people with lupus continue to have inadequate responses to these drugs [88]. Due to the possible clinical benefits for SLE, cellular treatments, and more especially MSCs, are disciplines of significant attention [13]. Steroid medications, a drug called azathioprine, and methotrexate are some of the immune‐suppressing drugs now used to treat SLE, while new therapeutics like rituximab monoclonal antibodies and immunomodulators like abatacept are among the numerous biological products currently undergoing clinical trials [89].

Recent years have seen a rise in interest in MSC treatment for immune‐mediated illnesses, including SLE. This treatment may reduce the signs and symptoms of refractory SLE by encouraging the proliferation of Th‐2 and Treg cells and repressing the activity of B‐lymphocytes, Th‐1, and Th‐17. However, some SLE patients have also claimed that MSC treatment is unsuccessful. This may be because of MSC‐ or patient‐related parameters. Thus, more investigation is needed to support the medical applications of MSCs [90]. It has been established that malfunctions in MSCs play a role in the onset of SLE [91].

Multiple investigations have shown that MSCs inhibit lymphocytic B cell development and expansion, hence reducing SLE incidence. Human gingival‐derived MSCs ameliorated proteinuria and histological grades of nephritis in a mouse model of lupus‐related nephritis by suppressing B cell function through the CD39/CD73 axis in vitro and in vivo [92]. By inhibiting the PD‐1/PD‐ligand system, MSCs may prevent lymphocytic B cells from developing into plasma cell populations [93]. Human BM‐MSC injections reduced glomerulonephritis, self‐reactive antibody production, proteinuria, and mortality in NZM/W F1 mice. The growth of follicular Th cells was hindered from accomplishing this [94]. By preventing plasmacytoid DCs from maturing and producing IFN‐α, MSCs may lower SLE severity. Human BM‐MSCs reduce inflammation of the renal system in mice with nephropathy induced by the Adriamycin antibiotic [95]. When given to a mouse lupus model, MSCs suppressed excessive inflammation and promoted the growth of B‐regulatory cells that produce IL‐10 [96]. MSC derived from the human UC injection decreased blood levels of Th17 and boosted levels of Treg cells in individuals with SLE [97].

The transplantation of allogeneic MSCs has been proven to be beneficial in a number of preliminary Phase 1 and Phase 2 studies; however, more research into the effects of this treatment on SLE over the long term is necessary [98]. It has also been shown that combining MSC transplantation with immunosuppressive treatment results in a considerable improvement in the patient's clinical condition. Additionally, its effectiveness primarily depends on the dosage, and regular administration may both slow the course of the illness and lower the likelihood of repeated episodes. However, to determine the optimum dosage and numerous infusion times, it will be necessary to conduct tests on a large scale [99].

### 3.2 MSCs in RA

RA is a kind of autoimmune illness that may last a person's whole life. It is characterized by inflammation in the synovial tissue of the joints as well as deterioration of the articular cartilage [99, 100]. Patients with RA have a higher chance of developing atherosclerosis, which may result in cardiovascular difficulties, which pose a significant danger to human health and life [101].

The primary goal of the current RA treatment choices is to achieve a clinically minimum disease activity score, and these alternatives include medications, physical therapy, surgical procedures, and other non‐pharmacological treatments [102]. Anti‐inflammatory medications, corticosteroids, and synthetic disease‐modifying anti‐rheumatic medicines such as methotrexate, sulfasalazine, hydroxychloroquine, and leflunomide are the standard therapies for RA. Biological disease‐modifying antirheumatic medicines are another kind of therapy. RA sufferers now have access to a greater variety of treatment options than in years past because of ongoing research and development efforts [103]. Typical treatments for illness provide only temporary alleviation from symptoms. Multiple strategies are used regularly as treatment for RA; unfortunately, neither of them can entirely alleviate the ailment. Therefore, RA treatment has to take a different approach. Because of this, researchers across the globe are trying to find new ways to manage RA and other inflammatory diseases at the cell level [104].

RA is a kind of immune disorder that is triggered not just by lymphocytic T‐ and B‐cells but also by certain inflammatory mediators, proteases, and additional factors that play a role in its origin [40]. Since MSCs are known to use cell types such as DCs, T‐ and B‐lymphocytic cells, and NK cells to regulate the release of cytokines [105], using them for the treatment of RA is seen as an intriguing possibility because these cells can mediate the pathological process of this autoimmune disorder [104]. Tissue injury healing is only one of the many functions associated with MSCs, including regulation of the surrounding area, activation of internal precursors via interactions between cells, and the discharge of different substances. Tissue reconstruction and rebuilding are aided by the many cytokines and growth factors that MSCs may create [106, 107]. Because of these features, MSCs are a promising treatment agent for RA [99]. In a previous study, to alleviate the symptoms of nine patients with severe RA, autologous BM‐MSCs were employed [108].

There have been close to 100 studies released on the subject of the development and verification of preliminary research models for the disease of RA. These models reveal promising patterns for the clinical use of MSC‐based therapies. Studies based on MSCs have shown, in a substantial number of preliminary models, that they can slow down the course of RA. Therefore, individuals who fail to respond effectively to the drug‐based therapies that are considered the standard level of treatment may benefit from MSC‐based therapies [109].

### 3.3 MSCs in Ankylosing Spondylitis (AS)

AS is a persistent, chronic form of inflammatory arthritis. AS typically affects the spinal joints, sacroiliac joints, and the soft tissues around them, such as tendons and ligaments. Inflammatory mechanisms linked to AS cause bone erosion, new bone growth, and ankylosis in the spine, which causes terrible pain, decreased spinal movement, and stiffness [110]. Numerous clinical signs and symptoms are expressed by AS; however, the most prevalent ones are persistent back pain and worsening spinal stiffness. Additionally, AS is linked to several kinds of joint problems, including finger, attachment point, peripheral joint, sacroiliac, and spinal joint. The advancement of the illness causes serious consequences including reduced spinal mobility, aberrant posture, hip discomfort, finger inflammation (sausage finger), attachment point inflammation, and peripheral arthritis, which severely disrupts the patient's regular life [111, 112]. Inflammatory bowel disease (IBD) [113], acute anterior uveitis [114], and psoriasis [115] are the most prevalent extra‐articular symptoms of AS. According to studies, AS also raises the risk of cardiovascular illness, pulmonary problems, as well as vertebral fractures via systemic inflammation, and reduced spinal mobility [116, 117].

At present, there are only a few effective therapies for AS, which are grouped into three categories: pharmacological [118, 119, 120, 121, 122], surgical [121, 122], and physical therapy and exercise [123]. Unfortunately, the existing treatment for AS is unable to stop the disease's progression or correct the structural damage to the spines or other joints [124, 125, 126, 127]. Substantial immunomodulatory molecules generated by MSCs, such as PGE2, TGF‐β, and HLA‐G5, inhibit the immune system by preventing DC activation and promoting the development of Treg cells. The proliferation and activation of effector T cells, including T‐helper 1 (Th1), T‐helper 17 (Th17), and cytotoxic T lymphocytes (CTL), that play a role in the pathogenesis of AS, are also inhibited by MSCs. Additionally, MSCs' production of IDO and PGE2 triggers the transition of macrophages from the pro‐inflammatory (M1) to the anti‐inflammatory (M2) phenotype [128, 129, 130, 131]. Consequently, MSC therapy might be used to enhance the management of AS.

While this is going on, several clinical and preclinical trials have been carried out to study the use of stem cells in AS patients and positive outcomes have been shown regarding the safety as well as the effectiveness of MSCs [132, 133]. Wang et al. [133] studied the safety and activity of allogeneic BM‐MSC intravenous infusion and showed that MSCs are safe and possess a promising therapeutic effect on active AS patients who do not respond to conventional pharmacological drugs. Li et al. [132] conducted an analogous trial and found that after receiving UC‐MSC infusion, all patients showed pain alleviation and reduction in scores indicating the severity and activity of AS. However, the common limitation in these trials is the reduced number of patients. On the other hand, several preclinical trials have been conducted on animal models and cell lines to evaluate the effectiveness and possible mechanisms and pathways of MSC the in correction of disease conditions of AS [134, 135, 136, 137, 138, 139, 140, 141, 142, 143, 144].

### 3.4 MSCs in OA

As the most prevalent type of arthritis, OA goes by a few other names: degenerative joint disease, or “wear and tear” arthritis. Hands, hips, and knees are the most common sites of this condition. Pain, stiffness, and edema are the results, making it the greatest cause of disability among the elderly. As a result, some individuals become disabled and are unable to do their normal daily activities or hold down a job [145]. Damage to articular cartilage, synovial membrane inflammation, and skeletal remodeling are all hallmarks of this condition [146].

Around 10%–15% of the human population is affected by OA [147]. There are several risk conditions, among them age, gender, history of trauma, overweight, and heredity. OA has not only displayed its greatest global prevalence [148] but has also caused many industrialized nations to endure huge financial losses since it is a disabling condition [149, 150]. At present, there is no perfect special medicine utilized for the therapeutic management of OA [151]. In the initial phases of OA, treatment often consists of alleviating pain, lifestyle moderation, and shedding pounds. A high tibial osteotomy is a surgical procedure that may benefit patients with mild OA of the knee [152]. For severe OA, replacement of a damaged joint is a common treatment option [153]. The present treatment for OA is mostly focused on reducing the severity of symptoms rather than stopping the disease progression, and long‐term cures have yet to be found [154].

Producing a functional substitute for natural cartilage is the primary objective and greatest difficulty of cartilage tissue repair [155, 156]. Cell therapy and rejuvenation treatments, especially those based on stem cell technology, may 1 day be all that's required to combat OA and help with joint repair [154, 156]. BM transplantation and MSCs derived from adipose tissue have emerged as the most popular treatment options and regenerative methods for OA [157]. When considering the treatment of degenerative joint problems, MSCs seem to provide some significant advantages over chondrocytes (Cartilage cells). They are easier to culture, proliferate at a faster rate, and have the potential to specialize in all of the tissues found inside the joint. In addition, it would seem that the paracrine activity is especially helpful in alleviating the symptoms of disease. MSCs, with their anti‐inflammatory and immunomodulatory properties, play an essential function in the process of directing the reconstructive reaction of damaged tissues of the joint [153, 158, 159].

Because of their potential to differentiate into chondrocytes, their capability to prevent chondrocyte suicide, and their potential to slow the degenerative condition as a whole, MSCs have shown promise as a treatment for OA [160]. In addition to suppressing T cell proliferation and blocking the respiratory burst in neutrophilic cells, these cells also produce cytokines and chemokines, which have an effect on the defense system's activity. Environmental factors disrupt the balance between MSCs' inflammatory and anti‐inflammatory properties [159]. Paracrine stimulation of the local surroundings is the primary way in which MSCs affect the regeneration procedure for OA joints. It has been discovered that MSCs stimulate cell renewal by secreting paracrine signals [153].

Multiple clinical studies have shown the potential therapeutic usefulness of MSCs for treating immunological and inflammation‐related illnesses [161, 162]. BM‐MSCs produce compounds with immunoregulatory and anti‐inflammatory properties [163]. Tissue injury triggers the recruitment and activation of regional tissue precursor cells, which have a greater capacity to influence the body's defenses. Therefore, because of their unique immunological characteristics and functions, MSCs effectively suppress immuno‐inflammatory reactions and promote tissue renewal [164]. An increase in the cells of the immune system in the synovium, specifically monocytic cells, macrophages, and eventually lymphocytic T‐cells, is a hallmark of OA. The appearance of lymphocytic B cells, DCs, mast cells, NK cells, and granulocytic cells is also seen in OA synovia [165]. Hypo‐immunogenic MSCs were thought to shield essential tissues from the effects of immune responses launched against invaders. Recent research, nevertheless, has shown that MSCs may be immune evasive. Allogeneic transplantation of MSCs could fail to have a promising future if MSCs are just “immune evasive” instead of “hypo‐immunogenic,” since the body's immune defense could ultimately recognize these cells as alien and attack them. There has to be further investigation into this matter, however [166]. The absence of a negative immunological reaction after delivery of allogenic MSCs remains a major benefit [167].

The difficulty of cartilage restoration is evident from the fact that no one has yet succeeded in restoring a physically and mechanically functional cartilage surface [168]. The use of mesenchymal cells in therapy is not risk‐free. Scar tissue development, immunological responses in the context of an allogeneic transplant, the cartilage's hyaline substance being replaced by bone, and the proliferation of cells that don't interact well together are all potential adverse effects [159, 169, 170].

### 3.5 MSCs in Osteoporosis (OP)

In OP, bone mass and microstructure deteriorate, making bones more prone to breaking and fracture than in healthy people [171]. Symptoms of OP do not appear until the disease has progressed for years, making it difficult to identify until the disease has severely limited a person's everyday life due to fractures. Individuals with osteoporotic fractures often end up bedridden and in a critical condition. Fractures are very dangerous since they may cause serious injuries and even death [172].

OP is now managed with a combination of pharmaceuticals and therapeutic exercise. However, finding new effective strategies to combat OP is urgently required due to the complexity of its cause and therapy, as well as the enormous bad effects of current drugs. OP patients have had reason for hope thanks to advances in MSC therapy in recent years [173].

Adjuvant therapies and pharmaceutical medication are now used for both the avoidance and management of OP. Suppression of osteoclasts (reduction in bone degradation) and encouragement of osteoblasts (increase in bone creation) are the two primary mechanisms by which medicines exert their beneficial effects on patients. The anti‐sclerostin monoclonal antibody romosozumab has a bidirectional mechanism of regulation, decreasing bone loss while increasing the creation of bones. Medications like denosumab and bisphosphonates may prevent bone breakdown, while teriparatide and abaloparatide can speed up the bone‐building process [174]. Calcium and vitamin D supplementation, as a non‐pharmaceutical therapy, has been prescribed for people with a higher likelihood of OP due to low vitamin D and calcium consumption and the onset of the menopause period [172, 175, 176].

A unique class of RNA molecules known as circular RNAs (circRNAs) have been shown to play important roles in a wide range of biological and pathological processes, including those that are directly relevant to bone health and disease [177]. Unexpectedly, circRNAs are also believed to play a role in OP control, including the disease's development and therapeutic approaches. Recent research has shown that circ_0005564 actively contributes to OP by raising mRNA concentrations of osteogenic differentiation biomarkers such as Runt‐related transcription factor 2(RUNX2), osteopontin, and osteocalcin [178].

There is a constant balance between bone production prompted by osteoblasts and bone resorption facilitated by osteoclasts, a process known as bone remodeling [179]. There are several variables, including growth regulators, hormones, cytokines, electrokinetic stimulation, and more, that influence and regulate the process of bone remodeling. MSCs, as the progenitor cells of osteoblasts, are crucial to the process of bone rejuvenation [180, 181]. Cell‐based regeneration treatments can potentially be effective in the treatment of OP by controlling bone loss, reducing fracture susceptibility, and increasing diminished mineral content. Boosting stem cell functions (including their growth and transformation into bone‐forming cells) and increasing precursor stem cell numbers achieve these goals [172, 182, 183]. Stem cells, particularly MSCs, have the potential to induce the regeneration of bone tissue by secreting physiologically active molecules. These substances include IGF‐1, TGF‐β, VEGF, angiogenin, HGF, and IL‐6. MSCs are especially important for this process [184, 185, 186].

Exosomes, which are tiny vesicles released by cells, offer advantageous properties such as significant permeability, minimal toxic effects, and efficient targeting. As a result, exosome treatment has emerged as a promising area of investigation for OP. When it comes to treating OP and bone fractures, bone reconstruction is a key modality. New bones with improved vascularization, biological mechanics, and histology may be prompted by exosomes released by MSCs [187]. Exosomes generated by MSCs are efficient variables whose effects on preventing the loss of bones and increasing skeleton‐building mechanisms (during the phases of bone formation, formation of osteoclast, and angiogenesis) have been shown in vitro and in vivo [188, 189].

MSC‐derived extracellular vehicles, also known as MSC‐EVs, provide an approach for cell‐free MSC treatment. In contrast to their mother cells, MSC‐derived EVs have a greater medicinal value while also posing a reduced risk of developing cancer. In vitro studies have shown that MSC‐EVs stimulate the process of osteogenesis and inhibit osteoclastogenesis, while in vivo studies have shown that they postpone the progression of OP. Recent years have seen significant advances made in the development of cell‐free stem cell treatments using MSC‐EVs. If successful, this line of investigation may 1 day become a viable treatment option for OP [190].

The transplantation of MSCs has the potential to restore an appropriate equilibrium between bone creation and loss, enhance osteoblast development, and inhibit osteoclast activity. The use of mesenchymal stromal cells (MSCs) in the management of OP has shown promise in clinical trials, with data suggesting that transplantation of these cells may improve the differentiation of osteogenic cells, boost the density of bone minerals, and arrest the progression of OP [1, 191]. In the meantime, modern methods such as modifying genes, targeted alteration, and co‐transplantation offer potential ways to increase the clinical impact and effectiveness of MSCs. Additionally, there are now clinical investigations being conducted for treating OP using MSC treatment. These trials will help cover what is lacking in medical research. Even though MSCs have shown promise as a treatment for OP, there are still pressing concerns about their safety, the effectiveness of transplants, and the need for the production method to be standardized [191].

### 3.6 MSCs in Sjogren's Syndrome (SS)

SS, also known as Sicca syndrome, is a kind of persistent immune‐mediated inflammation that occurs primarily in females. This condition influences the exocrine glands, leading to symptoms including dryness in the mouth (hyposialia or asialia) and dryness of the eye (xerophthalmia). Dental decay, periodontal illness, and fungus diseases are more likely to occur in those with SS‐induced hyposialia [192]. Other non‐glandular organs, including the layer of skin, may also be damaged, and the person's overall level of living gets worse as a result. SS may be diagnosed on its own or as a subsequent symptom of other autoimmune‐related conditions, including RA and SSc [193, 194]. It may be worsened by a variety of systematic consequences (such as lung disease, renal damage, and lymphoma) [195].

Disease‐modifying anti‐rheumatic medications like glucocorticoids are the gold standard for treating SS, but newer biotherapeutic techniques are making use of antibody therapy and blocking receptors that mediate inflammation to get better results. Targeting signaling and molecular mechanisms associated with lymphocytic B‐cells and Th17 have gained widespread popularity as a potential treatment for SS due to their prominent involvement in the disease's etiology. Improving outcomes and standards of living for SS sufferers is possible via the application of immunological blockers in the treatment of negative consequences. For instance, B lymphocyte‐activating factor (BAFF) receptor antagonists safeguard against lymphoma of B‐cells in addition to preventing inflammatory lesions; nonetheless, such treatment approaches are uncommon [195, 196, 197, 198].

SS is a syndrome where lymphocytes infiltrate the exocrine glands [199]. Based on research findings, it seems that BM‐MSCs in SS sufferers have impaired immunological activity, which may explain why SS develops in these individuals [200]. Higher expansion, immune system regulation, and the ability to differentiate in multiple directions are hallmarks of MSCs, which allow them to promote the regeneration of tissues while also suppressing the development of certain immune cell types, the release of pro‐inflammatory mediators, and the generation of antibodies. This has led to the development of an innovative approach for the treatment of SS, which is the application of MSCs [99].

Some studies demonstrated that MSCs were able to exert their curative benefits because of their immunomodulatory properties. Among these properties were the upregulation of Treg cells and the expansion of Th2, as well as the downregulation of Th17 and inflammatory reactions in follicular Th cells. Also, MSCs have been found in a number of studies to be effective in preventing the expansion of CD4+ T‐lymphocytes in non‐obese mice suffering from diabetes [201]. Another study reported that the use of UC‐derived MSCs has been shown to considerably boost the rate of salivary production, relieve manifestations of disease, and reduce inflammatory conditions. As a result, autologous MSC transplantation is anticipated to be employed as an innovative therapy option that is both highly successful and completely risk‐free [99].

Labial gland mesenchymal stem cells (LGMSCs) have recently been found and have shown higher efficiency [202]. In a mouse model of spontaneous SS, when the salivary glands were treated with exosomes made from LGMSCs (LGMSC‐Exos), they started working again. Co‐culturing LGMSC‐Exos with a patient's peripheral blood mononuclear cells (PBMNCs) in vitro has shown promise for treating SS. CD38 + CD27 + CD20‐CD19+ plasma cell percentages within PBMNCS were drastically lowered. Additional studies revealed that microRNA‐125b, which was generated from LGMSC‐Exo, affected the plasma cell population of the SS by specifically attaching to its targeted genes [203].

### 3.7 MSCs in Crohn's Disease (CD)

CD is a chronic, idiopathic IBD marked by intermittent lesions, patches alternate with healthy rejoin, and transmural inflammation that might influence the whole gastrointestinal system from the mouth to the anus [204, 205]. Common symptoms include severe abdominal pain, diarrhea, nausea, vomiting, weight loss, and occasionally chills or fever. The disease's characteristic pattern is one of remissions and flares. sadly, CD has no cure, and the majority of patients need at least one surgical excision [206]. Like other inflammatory and immune diseases, available therapy includes anti‐inflammatory, immunosuppressant, immunomodulators, monoclonal antibodies as well as biological therapy [207]. Medical treatment aims to achieve steroid‐free clinical and endoscopic remission to avoid complications and surgery [204].

Previous and current clinical trials employing MSCs for the treatment of CD have shown that this innovative therapy has the potential to induce and maintain full and long‐term remission of symptoms when standard medications have failed [208]. The exact mechanism of MSC in the case of CD is not fully understood, however, it has been believed that MSC could stimulate and accelerate the healing process of complicated fistula and produce immunomodulatory effects through interaction with immune cells, cytokines production and regulation of pro/anti‐inflammatory cytokines [209, 210, 211, 212]. After reviewing published studies, we found that the available clinical and preclinical studies are conducted for the management of fistulizing disease or active luminal disease [213, 214, 215, 216]. Several clinical trials in different phases demonstrated the promising effect of MSCs in the healing of fistulizing CD [210, 217, 218, 219, 220, 221, 222, 223, 224, 225, 226]. Most of these studies demonstrated the local effect [210, 218, 219, 220, 221, 222, 226] and others showed the effect of IV injection [217]. Additionally, Vieujean et al. [227] proved that MSC injection in CD stricture provides promising benefits and produces a partial and complete resolution of stricture. Moreover, they conducted another study [228] and demonstrated the same beneficial effect of MSC in non‐passable stricture. However, some occlusions were detected during the follow‐up, and they concluded that the result of CD stricture may be improved by combining the benefits of MSCs with the known effects of endoscopic balloon dilatation.

### 3.8 MSCs in Fibromyalgia (FM)

FM is a chronic neurologic disease that affects 2%–8% of the global population [229]. It is a complex pathophysiological condition characterized by chronic and generalized pain throughout the body. Persistent and severe generalized pain is the first symptom which is accompanied by spontaneous pain, cold allodynia, and thermal hyperalgesia, as well as exhaustion, sleep disorders, and depression with subsequent reduction in quality of life [230, 231]. The exact pathophysiology is not clear until now. Nevertheless, a large number of studies revealed the implementation of the central nervous system, evidenced by impairments in inhibitory pain pathways [232]. Furthermore, peripheral nervous system abnormalities such as pro‐inflammatory cytokine production and small nerve fiber dysfunction may have a role in disease development [233, 234]. Unfortunately, available treatment is a challenge as it provides symptomatic management only [235]. Numerous investigations showed that BM‐MSCs offer therapeutic potential for alleviating chronic pain [236, 237]. It is thought that MSCs could alter this disease condition through the reduction of inflammation and the demyelination process of nerves [238].

A recent and soul study that demonstrated the effect of MSCs in FM was conducted by Mekhemer et al. [239] They conducted their study on an experimental rat model. Rats were randomly divided into three groups; the control group, received 0.5% acetic acid vehicle, the FM group, received 1 mg/kg/day reserpine for induction of FM, and the FM‐BMSC group, which received 2 × 10^6^ BM‐MSCs after induction of FM. All groups of rats were killed 7 days following the BM‐MSCs injection, cerebral cortices were harvested for histological and biochemical analysis, along with physical and behavioral evaluations. According to the result of that study, the FM‐BM‐MSC group experienced an improvement in behavioral and physical assessment, reduction in pro‐inflammatory cytokines and inflammatory mediators as well as improvement in the histological picture, compared to other groups. Moreover, BM‐MSCs exhibited a neurogenesis activity that participated in the final beneficial outcomes.

### 3.9 MSCs in SSc

SSc is a persistent immune‐mediated illness that is identified by higher levels of production and accumulation of extracellular matrices in the skin and the interior of the body. The undesirable accumulation of collagen in the skin along with different tissues leads to the malfunction of various organs, and the outcome is not good for people whose lungs, hearts, or kidneys are involved in the condition [240]. Scleroderma is divided into two primary kinds, local scleroderma and SSc, based on the degree to which the skin is affected [241, 242]. Individuals with SSc have a much higher hospitalization and death rate than those with local scleroderma because of the widespread involvement of several systems inside the body [241].

The symptoms of SSc include fibrosis of internal and external tissues, inflammatory conditions, and vasculopathy. Scleroderma‐associated interstitial pulmonary disorder, high blood pressure in the lungs, and cardiovascular disorders are the major causes of mortality in people with serious SSc [243]. Scleroderma‐related interstitial pulmonary disease has been studied and treated with a number of anti‐inflammatory and anti‐fibrotic therapies. Treatments like these mostly serve to maintain the current situation or delay the progression of interstitial lung disease, but they do nothing to enhance pulmonary function or fibrotic interstitial lung disorder [244]. There is currently no effective or safe therapy for SSc, and fibrosis cannot be reversed [245].

Allogenic transplantation of HSCs has been a significant advancement in the treatment of resistant SSc in the last 10 years [246, 247]. Despite this, HSC allogeneic transfer is not recommended for individuals who have extensive visceral affection. Immunosuppression, antifibrosis, and proangiogenic properties of MSCs suggest that they might constitute an effective new therapy for the management of SSc [248].

MSCs have the ability to develop into bone cells, cells that make muscles, and endothelium cells. MSCs taken from individuals with SSc have been found to have unusual functional behaviors in relation to MSCs taken from normal subjects. These undesirable functions include impaired endothelial cell differentiation and higher levels of TGF‐β and VEGF, which could potentially have a significant role in the progression of fibrosis in SSc [249, 250]. In light of these results, autologous MSC transplants seem to have considerable potential as a treatment for SSc [99].

Whatever the cell source, major histocompatibility complex (MHC) matching, or application method, therapy based on MSC has been shown to be secure and efficient in several preliminary research investigations. MSCs have been shown to reduce inflammation and stop fibrosis in both bleomycin‐ and Hypochlorous acid (HOCl)‐induced mouse models of SSc. Using a mouse model of SSc, it was found that MSC‐based treatment effectively decreased dermal and pulmonary fibrosis [251, 252, 253, 254]. MSCs reduced dermal and pulmonary fibrosis in mouse HOCl‐induced SSc models by improving the remodeling of the extracellular matrix, lowering the amount of inflammatory cytokines, strengthening the body's defenses against antioxidants, and lowering the amount of anti‐Scl‐70 self‐antibodies in the serum of the mouse that was handled [253]. Based on these encouraging preliminary findings derived from mouse models of SSc, it seems that therapy with MSC‐base might be effective for SSc in the clinic [248].

A long‐period retrospective study with 9 years of follow‐up showed that all of the patients' skin symptoms and serological indices got better. This showed that MSC transplantation is an effective and safe way to treat SSc, while the deaths of six patients were not related to the MSC transplants [255]. Clinical investigations of MSC‐based treatment for SSc have begun after extensive in vivo and in vitro preliminary research [248]. Because of the limited number of instances and the variety of individuals involved, it is not possible to draw conclusive and trustworthy conclusions on the effectiveness of an MSC transplant for individuals with SSc [256, 257]. Even though research and clinical studies have shown that MSC‐based treatments are effective and safe, and this encourages more research and development of MSC‐based therapies for the treatment of SSc [257, 258, 259].

MSCs, like many other kinds of cells, produce external vesicles from the endosomal space. These vesicles, which have a layer of phospholipids surrounding them, are essential for cell interactions [260, 261, 262]. MSC transplantation has a number of issues that still need to be handled, such as the invasive techniques used to isolate cells, as well as effectiveness and safety problems [263, 264]. Since most of the medical benefits of MSCs have been linked to their paracrine signals, using MSC‐EVs as a “cell‐free” treatment may be an alternative that is similar to MSC treatment and may help get around some of the problems with MSC‐based therapies in the medical setting [265, 266]. MSC‐EVs are believed to provide a number of benefits over the original MSCs [248]. Among these benefits is the fact that they are not very immunogenic and have a reduced capacity to induce initial and acquired immunity in situ after autologous injection [255, 267, 268]. Due to their diminutive size compared to the original cells, MSC‐EVs may be transported more effectively to the lung tissue and other target organs [269]. When compared to their original MSCs, MSC‐EVs have higher levels of safety and minimal negative consequences [261]. Several potential risks associated with MSC transplants, such as ossification or accumulation of calcium in tissues, may be avoided with the application of EVs [270].

### 3.10 MSCs in Dermatomyositis (DM) and Polymyositis (PM)

DM and PM are the critical types of idiopathic inflammatory myopathies [271]. They are characterized by proximal skeletal muscle weakness and apparent skin symptoms. Moreover, they might impact other organs, including the lungs, heart, GIT, and kidneys [272, 273]. Despite their clinical presentations being different, they are common in symmetrical and proximal muscle weakness. Physical therapy, immunosuppression, drug monitoring for side effects, and complication avoidance are the cornerstones of DM and PM treatment [274]. Until now, the etiology is unknown. Since cellular dysfunction related to Th cells is crucial for the incidence and progression of PM or DM, several studies have demonstrated that Th cells are implicated in the pathogenesis of DM/PM [275]. Consequently, MSCs may offer a novel therapeutic approach to the management of DM and PM. Numerous research studies have shown that this strategy produces beneficial outcomes.

According to Wang et al. [276] study, allogeneic BM‐MSC or UC‐ MSC at a dose of 1 × 10^6^ cells/Kg IV infusion was given to 10 patients with drug‐resistant DM/PM or aggressive systemic symptoms. Serum creatine kinase (CK) levels improved, and some patients with interstitial lung disease and skin ulcers also had improvements. For recurrence, several patients needed an additional MSC dose. According to the authors, a controlled prospective study is needed. In another report, at a 3‐month follow‐up, a 35‐year‐old female experienced dramatically better strength following a four IV infusion of autologous expanded adipose‐derived MSC [277]. In long‐term retrospective research by Liang et al. [255], IV infusion of 1 × 10^6^ cells/Kg body weight of MSCs led to improvement in symptoms and serological markers of patients after 9 years of follow‐up. This investigated the efficacy and safety of MSC in PM and DM, although 11 patients died for causes unrelated to transplantation. In another study by Lai et al. [275], DM/PM patients received IV infusion of 3.5–5.2 × 10^7^ UC‐MSCs, transplantation group, and the degree of improvement was compared with the control group, treated with glucocorticoids and immunosuppressants for 6 months. The study's findings revealed that both groups' CK levels had dramatically lowered, but the transplant group had better outcomes than the control group at various periods and had much superior lung function. After the transplant, one patient died, however, no transplant‐related problems happened. Furthermore, Lai et al. [275] found that INF‐γ levels considerably increased at 6 months following MSC infusion, although IL‐4 levels dramatically reduced at the same time, and IL‐17 levels similarly declined at 3 and 6 months following cell infusion. These results suggest that UC‐MSC transplantation in combination with glucocorticoid and immunosuppressive medication can promote immunological tolerance and alter immune network effects in DM/PM patients.

At present, just a few studies looking into PM/DM; therefore, large‐scale, randomized clinical trials are required to assess the long‐term efficacy and safety of MSC transplantation in PM/DM patients, taking into account the dangers of infections and tumors in addition to the ideal dose for transplantation.

## 4 Obstacles and Challenges With MSCs Transplantation Therapy

Transplantation of MSCs is an exciting new approach to treating rheumatic conditions. However, several significant hurdles must be cleared before they may be routinely used on patients. One of these obstacles is determining which donors and tissues would provide the highest‐quality MSCs for use in treating certain patients. There is a large inter‐donor variability in MSC quality [278, 279]. Lacking standard operating procedures for dealing with MSCs [280]. There is a significant amount of diversity in the quality of the cells, which is the primary barrier to the standard techniques of MSC [265, 281, 282]. Aggressive isolation techniques and time‐consuming cell culture methods [283]. Their ability to scale up is restricted due to MSCs' low rate of proliferation in vitro. MSC treatment often requires a substantial quantity of cells to exert its curative benefits [284]. Short‐term survival of externally introduced MSCs in vivo was observed [285, 286, 287]. Ineffective recruitment or adherence to the intended cells following systematic treatment, as well as decreased transplantation effectiveness [285, 288, 289, 290, 291]. Despite the fact that a great number of preliminary research investigations have shown its safety [292, 293], The widespread application of MSCs in healthcare settings must not be moved forward until their definite safety concerns, such as genetic disorders, unwanted proliferation of grafted MSCs, and tumor development potential in vivo, are considerably investigated [263, 294]. Even though MSC‐based treatment has shown positive outcomes in both preliminary and clinical investigations, these findings are inconsistent and even conflicting sometimes [295, 296]. Possibility of developing microthrombosis as a result of receiving MSC injections. According to the findings of preliminary research, the vast majority of MSCs administered intravenously get promptly stuck in lung parenchymal capillaries having a size smaller than that of MSCs [297, 298, 299, 300]. This phenomenon has the potential to generate multifocal lung atelectasis and thrombosis [264, 297, 301].

## 5 Conclusion and Future Perspectives

Due to their self‐renewal, differentiation multipotency, paracrine potentials, long‐term ex vivo development, and immunomodulatory activities, MSCs are a viable cell therapy and tissue repair alternative. Rheumatic illnesses are immune‐mediated and inflammation‐related disorders that can affect every system in the body and cause chronic pain, joint stiffness, fatigue, irreversible disability, and organ damage, lowering the quality of life. The main drawbacks of conventional treatment for these diseases are that there are few or no effective treatments, the treatment focuses on relieving symptoms, and there is no cure. Despite medical advancements, morbidity and disability remain high. MSCs' regenerative and immunomodulatory characteristics make them intrigued for treating rheumatic illnesses.

Recent research has demonstrated the promise of MSC‐based therapies for rheumatic diseases. MSCs are simple to separate from multiple sources, can develop quickly into large numbers for therapeutic application, have fewer ethical problems than ESCs, and have a reduced risk of teratomas than iPSCs. They move to injured tissue through chemoattraction, making them beneficial for many therapeutic applications. MSCs showed promise in treating rheumatic diseases like RA, SLE, SSc, OA, OP, AS, CD, DM, PM, FM, SS, and others.

MSCs in clinical practice must overcome various obstacles. These problems include MSC sources, characterization, standardization, safety, and effectiveness issues; aggressive isolation techniques and time‐consuming cell culture methods; a low rate of proliferation in vitro; short‐term survival of MSCs introduced from the outside in vivo; and ineffective recruitment or adherence to the intended cells after systematic treatment. Therefore, MSCs should not be widely used in healthcare until their safety problems, such as genetic abnormalities, undesired proliferation of transplanted MSCs, and in vivo tumor formation potential, are well explored. MSC treatment may help treat various rheumatic disorders such as Bursitis, Gout, Relapsing polychondritis, Juvenile idiopathic arthritis, Psoriatic arthritis, etc. Thus, future studies are needed to explain how to overcome these obstacles and maximize the benefits of MSC. Finally, translational research is essential for bench‐to‐bedside translation and clinical use in the near future.

## Author Contributions

Conception and design: Helal F. Hetta, Alaa Elsaghir, and Yasmin N. Ramadan. Writing – original draft preparation, data analysis, curation, and visualization: Helal F. Hetta, Alaa Elsaghir, Yasmin N. Ramadan, Abdulrahman K. Ahmed, Sayed A. Gad, Victor Coll Sijercic, and Mahlet S. Zeleke. Preparing of figures: Abdulrahman K. Ahmed, Sayed A. Gad, Victor Coll Sijercic, and Helal F. Hetta. Reviewing and editing: Helal F. Hetta, Alaa Elsaghir, Fawaz E. Alanazi, and Yasmin N. Ramadan. Acquisition of data for the work and revised the manuscript critically for important intellectual content: Helal F. Hetta, Alaa Elsaghir, Fawaz E. Alanazi, Mahlet S. Zeleke and Yasmin N. Ramadan All authors provided final approval of the version to be published. All authors have agreed to be accountable for all aspects of the work.

## Ethics Statement

The authors have nothing to report.

## Consent

The authors have nothing to report.

## Conflicts of Interest

The authors declare no conflicts of interest.

## Data Availability

All generated data are included in this manuscript.
